# Preschool-Aged Household Contacts as a Risk Factor for Viral Respiratory Infections in Healthcare Personnel

**DOI:** 10.1093/ofid/ofad057

**Published:** 2023-02-08

**Authors:** Zachary M Most, Ann-Christine Nyquist, Lewis J Radonovich, Maria C Rodriguez-Barradas, Connie Savor Price, Michael S Simberkoff, Mary T Bessesen, Derek A T Cummings, Susan M Rattigan, Charlotte Warren-Gash, Charlotte A Gaydos, Cynthia L Gibert, Geoffrey J Gorse, Trish M Perl

**Affiliations:** Pediatric Infectious Diseases Program, Children’s Health System of Texas, Dallas, Texas, USA; Division of Infectious Disease, Department of Pediatrics, University of Texas Southwestern Medical Center, Dallas, Texas, USA; Department of Infectious Disease, Children’s Hospital Colorado, Aurora, Colorado, USA; Division of Infectious Diseases, Department of Pediatrics, University of Colorado School of Medicine, Aurora, Colorado, USA; Respiratory Health Division, National Institute for Occupational Safety and Health, Centers for Disease Control and Prevention, Morgantown, West Virginia, USA; Infectious Diseases Section, Michael E. DeBakey Veterans Affairs Medical Center, Houston, Texas, USA; Department of Medicine, Baylor College of Medicine, Houston, Texas, USA; Division of Infectious Diseases, University of Colorado School of Medicine, Aurora, Colorado, USA; Infectious Disease Department, Denver Health Medical Center, Denver, Colorado, USA; Department of Medicine, Veterans Affairs New York Harbor Healthcare System, New York, New York, USA; Division of Infectious Diseases, New York University Grossman School of Medicine, New York, New York, USA; Division of Infectious Diseases, University of Colorado School of Medicine, Aurora, Colorado, USA; Medical Service/Infectious Disease, Veterans Affairs Eastern Colorado Healthcare System, Aurora, Colorado, USA; Department of Biology and Emerging Pathogens Institute, University of Florida, Gainesville, Florida, USA; Department of Epidemiology, Johns Hopkins Bloomberg School of Public Health, Baltimore, Maryland, USA; Department of Biology and Emerging Pathogens Institute, University of Florida, Gainesville, Florida, USA; Faculty of Epidemiology and Population Health, London School of Hygiene and Tropical Medicine, London, United Kingdom; Department of Medicine and Division of Infectious Diseases, Johns Hopkins School of Medicine, Baltimore, Maryland, USA; Medical Service/Infectious Disease, Veterans Affairs Medical Center, Washington, District of Columbia, USA; Department of Medicine, George Washington University School of Medical and Health Sciences, Washington, District of Columbia, USA; Division of Infectious Diseases, Allergy and Immunology, Saint Louis University School of Medicine, St Louis, Missouri, USA; Department of Epidemiology, Johns Hopkins Bloomberg School of Public Health, Baltimore, Maryland, USA; Division of Infectious Diseases and Geographic Medicine, University of Texas Southwestern Medical Center, Dallas, Texas, USA

**Keywords:** healthcare-associated infections, healthcare personnel, viral respiratory infections

## Abstract

**Background:**

Viral respiratory infections (VRIs) are common and are occupational risks for healthcare personnel (HCP). VRIs can also be acquired at home and other settings among HCPs. We sought to determine if preschool-aged household contacts are a risk factor for VRIs among HCPs working in outpatient settings.

**Methods:**

We conducted a secondary analysis of data from a cluster randomized trial at 7 medical centers in the United States over 4 influenza seasons from 2011–2012 to 2014–2015. Adult HCPs who routinely came within 6 feet of patients with respiratory infections were included. Participants were tested for respiratory viruses whenever symptomatic and at 2 random times each season when asymptomatic. The exposure of interest was the number of household contacts 0–5 years old (preschool-aged) at the beginning of each HCP-season. The primary outcome was the rate of polymerase chain reaction–detected VRIs, regardless of symptoms. The VRI incidence rate ratio (IRR) was calculated using a mixed-effects Poisson regression model that accounted for clustering at the clinic level.

**Results:**

Among the 4476 HCP-seasons, most HCPs were female (85.4%) and between 30 and 49 years of age (54.6%). The overall VRI rate was 2.04 per 100 person-weeks. In the adjusted analysis, HCPs having 1 (IRR, 1.22 [95% confidence interval {CI}, 1.05–1.43]) and ≥2 (IRR, 1.35 [95% CI, 1.09–1.67]) preschool-aged household contacts had higher VRI rates than those with zero preschool-aged household contacts.

**Conclusions:**

Preschool-aged household contacts are a risk factor for developing VRIs among HCPs working in outpatient settings.

## Teaser

In a secondary analysis of a randomized trial among healthcare personnel working in outpatient settings, having preschool-aged children at home was a risk factor for viral respiratory infections.

Viral respiratory infections (VRIs) are ubiquitous in humans, with illness ranging from no symptoms to life-threatening lower respiratory tract illnesses [1–3]. For healthcare personnel (HCP) who routinely come face-to-face with infected patients, VRIs can be prevented [4–7]. The coronavirus disease 2019 (COVID-19) pandemic has reinforced the potential dangers that HCPs face from occupational exposure to respiratory viruses. Infection from workplace exposure to VRIs negatively impacts HCP physical and mental welfare, contributes to burnout, and causes resignations [8, 9]. This reduction in the healthcare workforce increases stress on overburdened healthcare systems and negatively impacts patient safety [10].

HCPs are at increased risk for acquiring VRIs if they work in pediatrics [11] or perform aerosol-generating procedures [12]. Risk can be mitigated by workplace-based interventions, including wearing personal protective equipment [13]. Nonoccupational exposures, however, may account for most HCP infections. In a recent cohort of Canadian HCPs, sick contacts at home and in social settings were strongly associated with VRIs, whereas contact with patients with VRIs was not [7]. Additional studies have shown that younger age, children aged <15 years at home, and multiple children at home increase the risk of HCPs developing influenza infections [14–16].

Children, in particular those who are preschool-aged, have a much higher incidence of VRIs than adults [17, 18], and adults are more likely to develop VRIs when they live with children [19, 20]. Among HCPs with occupational exposure, it is unclear if exposure to children independently increases the risk for acquiring VRIs and whether this risk is important for respiratory viruses other than influenza, especially among HCPs with high influenza vaccination coverage. Therefore, the aim of this study was to determine if having preschool-aged children at home is an independent risk factor for developing VRIs among HCPs.

## METHODS

A post hoc analysis of the data collected from the Respiratory Protection Effectiveness Clinical Trial (ResPECT) was performed. ResPECT was a cluster randomized trial comparing the effectiveness of N95 respirators to medical masks for preventing influenza and other VRIs in HCPs who worked in outpatient settings. ResPECT was conducted over 4 influenza seasons (2011–2012 to 2014–2015) at 137 clinics and emergency departments (clusters) affiliated with 7 medical centers throughout the United States. The study protocol and primary study results have been published previously [21, 22].

HCPs were enrolled if they worked in outpatient settings where they encountered patients with acute respiratory illnesses, were ≥18 years old, routinely came within 6 feet of patients, worked full-time at the medical center, worked at least 75% of their time at 1 clinic, provided informed consent, and agreed to and passed N95 respirator fit testing. Each season, the recruiting process was repeated, and clinics and individual HCPs could enroll for >1 season. As demographics and exposures could change for participants between seasons, the unit of analysis for this study is each HCP-season. The intention-to-treat (ITT) population included all HCP-seasons enrolled with the intervention randomized at their clinic, and the per-protocol population only included HCP-seasons with at least 8 weeks of follow-up. The per-protocol population was used in the primary analysis for this study.

At enrollment, individuals completed a baseline self-administered questionnaire. The study remained active for 12 weeks each year during peak influenza incidence, as determined using the Above Local Elevated Respiratory Illness Threshold (ALERT) algorithm [23]. VRI symptoms were self-reported weekly in a diary. Each participant provided a combined nasal and throat sample by self-collection or research assistant collection while symptomatic and at 2 random times throughout the study period, as previously described [21]. Eligible signs/symptoms were coryza, temperature >37.8°C, lymphadenopathy, respiratory rate >25 breaths/minute, arthralgias/myalgias/body aches, chills, cough, diarrhea, dyspnea, fatigue, headache, malaise, nausea/vomiting, other gastrointestinal symptoms, sore throat, sputum production, or sweats. The specimens were tested by multiplex reverse-transcription polymerase chain reaction (RT-PCR) assays that included the following viruses: respiratory adenoviruses, 4 endemic human coronaviruses (hCOVs), human metapneumovirus, influenza A, influenza B, parainfluenza viruses 1–4, respiratory syncytial virus (RSV), and human rhinovirus/enterovirus.

In this cohort, the exposure of interest was the number of household contacts (HHCs) aged 0–5 years (preschool-aged) at the beginning of each season, as determined from the baseline questionnaire. To assess for a “dose-dependent response,” the number of preschool-aged HHCs was categorized into zero, 1, and ≥2. The primary outcome was the incidence rate of PCR-detected viral respiratory infections (PCR-VRIs). A PCR-VRI event was defined as detection of any of the above viruses by multiplex RT-PCR on a respiratory specimen, regardless of the presence of symptoms. If >1 virus was detected in the same specimen, these were each counted as unique PCR-VRI events. If the same virus was detected on a second specimen that was collected <21 days after the first positive result, it was considered the same infection and the second result was excluded. The incidence rate of PCR-VRIs for each of the viruses under investigation was evaluated as a secondary outcome. The follow-up time for each HCP-season, measured in weeks, was defined as the difference between the date that the clinic was activated in the study and last date of participation, with a maximum of 12 weeks per season.

All statistical analyses were completed in Stata version 16.1 software (StataCorp, College Station, Texas). HCP-seasons with missing data on the number of preschool-aged HHCs were excluded from the analysis. HCP-seasons with missing data on potential confounders (defined below) were excluded from the adjusted analysis.

The incidence rate ratio (IRR) of PCR-VRIs between HHC exposure groups was calculated using a mixed-effects Poisson regression model that accounted for clustering at the clinic level. The null hypothesis assumed no association between the number of preschool-aged HHCs and rate of PCR-VRIs and was tested using the likelihood ratio test (LRT) comparing models with and without the 2 dummy variables for preschool-aged HHC categories. A multivariable model was then created to adjust the effect for a priori confounders (age, sex, occupational exposure risk). No other variables were determined to be confounders in the analysis.

There were 3 planned sensitivity analyses. The first utilized only symptomatic PCR-confirmed respiratory illnesses, for which viral coinfections were counted as 1 event. The second included HCP-seasons in the ITT population with at least 1 week of follow-up time and no missing data on HHCs. The third only included HCP-seasons from individuals who participated in >1 study season and whose number of preschool-aged HHCs changed at least once between seasons. An additional post hoc analysis using acute respiratory illness regardless of PCR testing results was also completed.

### Patient Consent Statement

The ResPECT trial protocol was approved by the Human Subjects Review Board at the National Institute for Occupational Safety and Health (protocol number 10-NPPTL-O5XP). Additionally, the study was approved by the institutional review boards at each participating health system, analysis site, and storage site. All participants provided written informed consent to participate each season.

## RESULTS

**Figure 1.**
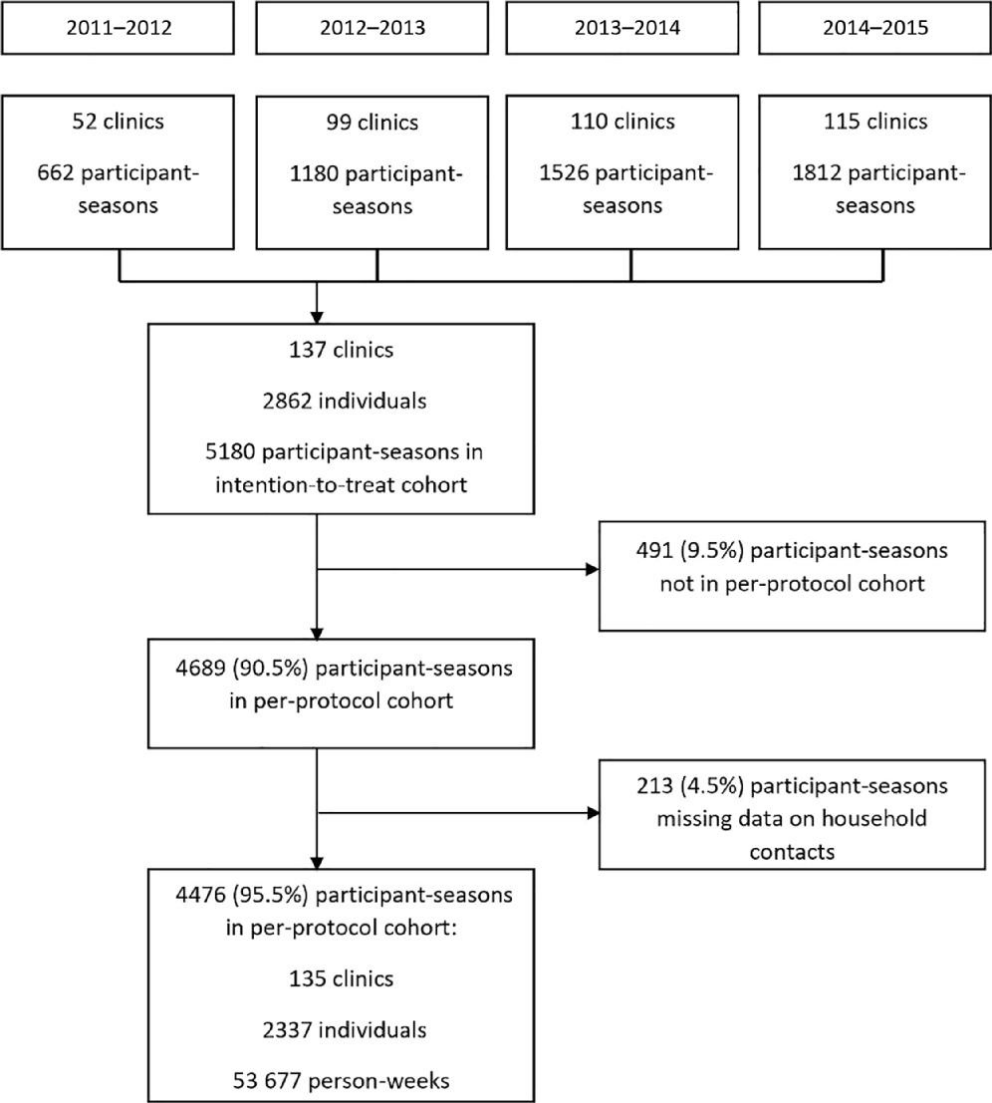
Flowchart showing participation in the Respiratory Protection Effectiveness Clinical Trial (ResPECT) and inclusion in this study over 4 seasons.

There were 2337 individuals who participated in 4476 HCP-seasons (1.9 seasons per individual) with a total of 53 677 person-weeks of follow-up (mean, 11.99 weeks per HCP-season; n = 17 [0.3%] withdrew early; Figure 1) after excluding HCP-seasons that had missing data on preschool-aged HHCs (Supplementary Table 1). Female participants (n = 3823 [85.4%]) who were between 30 and 49 years of age (n = 2444 [54.6%]; Table 1) accounted for the most HCP-seasons. Participants who self-identified as non-Hispanic White (n = 1973 [44.1%]) accounted for a plurality of HCP-seasons, followed by non-Hispanic Black (n = 1284 [28.2%]) and Hispanic (n = 689 [15.4%]). The most common underlying medical condition was lung disease including asthma (n = 458 [10.3%]). There were few active or former smokers (n = 369 [8.3%]). The majority received influenza vaccination (n = 3663 [82.3%]), but only 49.5% of preschool-aged HHCs had received influenza vaccination. Most study participants worked with adult patients exclusively (n = 2428 [54.2%]), followed by both pediatric and adult patients (n = 1037 [23.2%]) and pediatric patients exclusively (n = 1011 [22.6%]), and most worked in high-occupational-risk settings (n = 2680 [59.9%]). A plurality of participants were nurses (n = 1838 [41.1%]), followed by physicians (n = 486 [10.9%]). Most participants worked in primary care (n = 3115 [69.6%]), followed by emergency care (n = 1272 [28.4%]). The number of participating HCPs increased each season, reaching its maximum in 2014–2015 (n = 1544 [34.5%]).

**Table 1.** Baseline Characteristics of Healthcare Personnel (HCP) Participant-Seasons by Number of Preschool-Aged (0–5 Years) Household Contacts (N = 4476 HCP-Seasons)

Characteristic (Exposure in Participants)	Category/Grouping	Total (%)	No. of HHCs 0–5 y (Column %)			*P* Value^a^
			0	1	≥2	
Sex	Female	3823 (85.4)	2857 (84.6)	704 (89.5)	262 (84.0)	.002
	Male	653 (14.6)	520 (15.4)	83 (10.6)	50 (16.0)	
Age, y (n = 4470)	18–29	648 (14.5)	452 (13.4)	158 (20.1)	38 (12.2)	<.001
	30–39	1329 (29.7)	797 (23.6)	350 (44.6)	182 (58.3)	
	40–49	1115 (24.9)	851 (25.2)	189 (24.1)	75 (24.0)	
	50–59	1020 (22.8)	939 (27.8)	68 (8.7)		
	≥60	358 (8.01)	334 (9.9)	20 (2.6)	17 (5.5)^b^	
Race/ethnicity	NH White	1973 (44.1)	1551 (45.9)	271 (34.4)	151 (48.4)	<.001
	NH Black	1284 (28.2)	970 (28.7)	225 (28.6)	69 (22.1)	
	Hispanic	689 (15.4)	424 (12.6)	218 (27.7)	47 (15.1)	
	Asian	337 (7.53)	268 (7.9)	46 (5.8)	23 (7.4)	
	Other	213 (4.76)	164 (4.9)	27 (3.4)	22 (7.1)	
Smoker (n = 4440)	Yes	369 (8.31)	286 (8.6)	58 (7.4)	25 (8.0)	.57
	No	4071 (91.7)	3059 (91.5)	725 (92.6)	287 (92.0)	
Lung disease (n = 4457)	Yes	458 (10.3)	326 (9.7)	87 (11.1)	45 (14.5)	.02
	No	3999 (89.7)	3039 (90.3)	694 (88.9)	266 (85.5)	
Influenza vaccine (n = 4451)	Yes	3663 (82.3)	2719 (81.0)	676 (86.5)	268 (86.2)	<.001
	No	788 (17.7)	639 (19.0)	106 (13.6)	43 (13.8)	
Facial protection randomization	MM	2355 (52.6)	1783 (52.8)	402 (51.1)	170 (54.5)	.54
	N95 respirator	2121 (47.4)	1594 (47.2)	385 (48.9)	142 (45.5)	
Practice setting	Pediatric	1011 (22.6)	750 (22.2)	184 (23.4)	77 (24.7)	<.001
	Adult	2428 (54.2)	1910 (56.6)	369 (46.9)	149 (47.8)	
	Both	1037 (23.2)	717 (21.2)	234 (29.7)	86 (27.6)	
Occupational risk^c^	High	2680 (59.9)	2072 (61.4)	414 (52.6)	194 (62.2)	<.001
	Medium	536 (12.0)	366 (10.8)	133 (16.9)	37 (11.9)	
	Low	1260 (28.2)	939 (27.8)	240 (30.5)	8 (26.0)	
No. of HHCs 6–24 y	0	2358 (52.7)	1871 (55.4)	284 (36.1)	203 (65.1)	<.001
	1	1015 (22.7)	661 (19.6)	282 (35.8)	72 (23.1)	
	2	698 (15.6)	518 (15.3)	158 (20.1)	22 (7.1)	
	3	297 (6.6)	245 (7.3)	46 (5.8)	6 (1.9)	
	≥4	108 (2.4)	82 (2.4)	17 (2.2)	9 (2.9)	
Medical center	Johns Hopkins Health System	1561 (34.9)	1156 (34.2)	297 (37.7)	108 (34.6)	<.001
	Denver Health Medical System	929 (20.8)	623 (18.5)	227 (28.8)	79 (25.3)	
	VA New York Harbor Healthcare System	575 (12.9)	447 (13.2)	79 (9.91)	50 (16.0)	
	Michael E. DeBakey VAMC	454 (10.1)	378 (11.2)	57 (7.24)	19 (6.09)	
	Washington, DC VAMC	352 (7.86)	294 (8.71)	36 (4.57)	22 (7.05)	
	VA Eastern Colorado Healthcare System	348 (7.77)	291 (8.62)	46 (5.84)	11 (3.53)	
	Children's Hospital Colorado	257 (5.74)	188 (5.57)	46 (5.84)	23 (7.05)	
Season	2011–2012	531 (11.9)	386 (11.4)	100 (12.7)	45 (14.4)	.20
	2012–2013	1074 (24.0)	792 (23.5)	200 (25.4)	82 (26.3)	
	2013–2014	1327 (29.7)	1018 (30.2)	232 (29.5)	77 (24.7)	
	2014–2015	1544 (34.5)	1181 (35.0)	255 (32.4)	108 (34.6)	

A total of 2337 individuals contributed 4476 healthcare personnel–seasons of follow-up.

Abbreviations: HHC, household contact; MM, medical mask; NH, non-Hispanic; VA, Veterans Affairs; VAMC, Veterans Affairs Medical Center.

a Calculated by χ^2^ test between all 3 HHC groups.

b Age 50–59 years and ≥60 years cells merged due to sparse values to protect participant identities.

c Occupational risk scale: high, direct patient contact with performance of high-risk procedures (intubation, airway suctioning, nebulizer treatments, nasopharyngeal aspiration); medium, direct patient contact without high-risk procedures; low, minimal patient contact.

Overall, most participants had zero preschool-aged HHCs (n = 3377 [75.4%]), while 17.6% (n = 787) had 1 and 7% (n = 312) had ≥2. There were 1095 PCR-VRIs (612 symptomatic [55.9%]), with an incidence rate of 2.04 per 100 person-weeks. The incidence rate of PCR-VRIs increased among those with more preschool-aged HHCs. Accounting for clustering, among HCP-seasons with zero, 1, and ≥2 preschool-aged HHCs, the incidence rate of PCR-VRIs was 1.87 (95% confidence interval [CI], 1.74–2.02), 2.42 (95% CI, 2.12–2.76), and 2.68 (95% CI, 2.20–3.26) per 100 person-weeks, respectively. The rate of PCR-VRIs was 29% greater in those with 1 preschool-aged HHC compared to none (IRR, 1.29 [95% CI, 1.12–1.50]), and was 43% greater in those with ≥2 preschool-aged HHCs compared to none (IRR, 1.43 [95% CI, 1.16–1.76]; *P* = .0001). Utilizing only the 4470 HCP-seasons with complete data on covariates, the rates were similar (Table 2).

**Table 2.** **Rate of all Polymerase Chain Reaction–Detected** ^**a**^ **Viral Respiratory Infections in Healthcare Personnel (HCP) by Number of Preschool-Aged Household Contacts (n = 4470 HCP-Seasons)**

No. of HHCs Aged 0–5 y	No. of PCR-VRIs	Follow-up Time^b^	Incidence Rate (95% CI)^c,d^	Unadjusted Rate Ratio (95% CI)^d^	*P* Value^e^	Adjusted Rate Ratio (95% CI)^d^	*P* Value^e^
0	763	40 455	1.87 (1.74–2.02)	1.00 (ref)	.0001	1.00 (ref)	.003
1	230	9407	2.43 (2.13–2.77)	1.30 (1.12–1.50)		1.22 (1.05–1.43)	
≥2	101	3743	2.68 (2.20–3.26)	1.43 (1.16–1.76)		1.35 (1.09–1.67)	

The adjusted model included sex, age, and occupational exposure risk.

Abbreviations: CI, confidence interval; HHC, household contact; PCR-VRI, polymerase chain reaction–detected viral respiratory infection.

a PCR identified 13 respiratory viruses. Symptomatic and asymptomatic detections were included.

b Follow-up time in person-weeks.

c Incidence rate per 100 person-weeks.

d Accounts for clustering at the clinic level, using a mixed-effects Poisson regression model.

e Calculated using likelihood ratio test.

**Figure 2.**
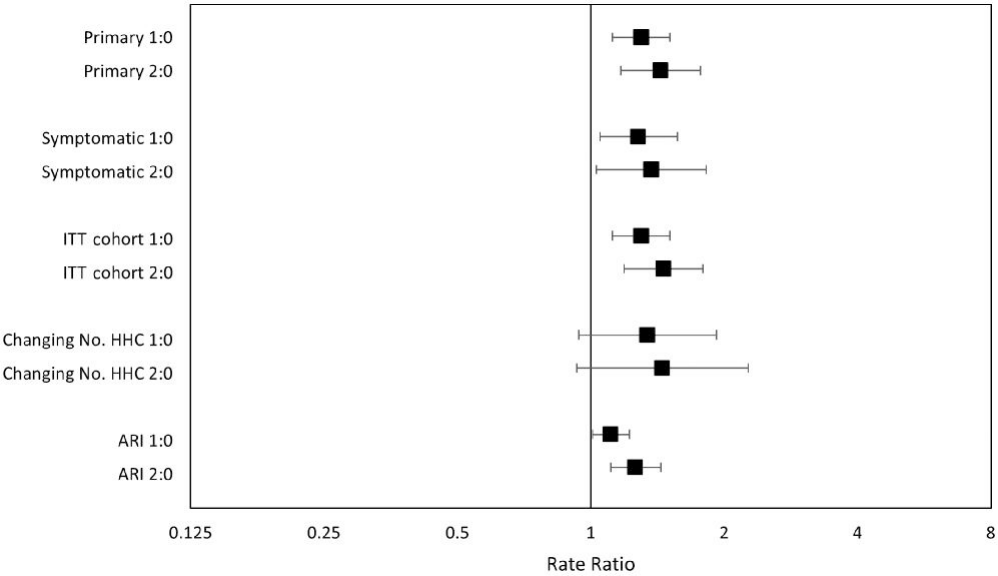
Sensitivity analyses with adjusted rate ratios for polymerase chain reaction (PCR)–detected viral respiratory infections in healthcare personnel (HCP) by number of preschool-aged household contacts (HHCs). For each analysis, “1:0” compares 1 preschool-aged HHC to zero, and “2:0” compares ≥2 preschool-aged HHCs to zero. “Primary” refers to the adjusted primary analysis and is shown for comparison to the sensitivity analyses. “Symptomatic” uses an alternate outcome definition that only included symptomatic individuals, and viral coinfections were counted as 1 event. “ITT cohort” (intention-to-treat) includes all HCP-seasons, but excludes those with missing HHC data (n = 228), missing age data (n = 6), and zero follow-up time (n = 278). “Changing No. HHC” only includes individuals who participated for >1 season and had their number of preschool-aged HHCs change at least once between seasons. “ARI” (acute respiratory illness) uses ARI regardless of PCR testing result as the outcome. Incidence rate ratios were calculated using a mixed-effects Poisson regression model that accounted for clustering at the clinic level.

In the adjusted model, there remained a strong association between having 1 and ≥2 preschool-aged HHCs and PCR-VRIs (IRR for 1 HHC, 1.22 [95% CI, 1.05–1.43]; IRR for ≥2 HHCs, 1.35 [95% CI, 1.09–1.67]; *P* = .003; Table 2, Supplementary Table 2). In all 4 sensitivity analyses, the point estimates of risk associated with preschool-aged HHCs were similar to the primary analysis (Figure 2 and Supplementary Table 3), although some analyses had fewer events, leading to less precise estimates with CIs crossing unity.

**Figure 3.**
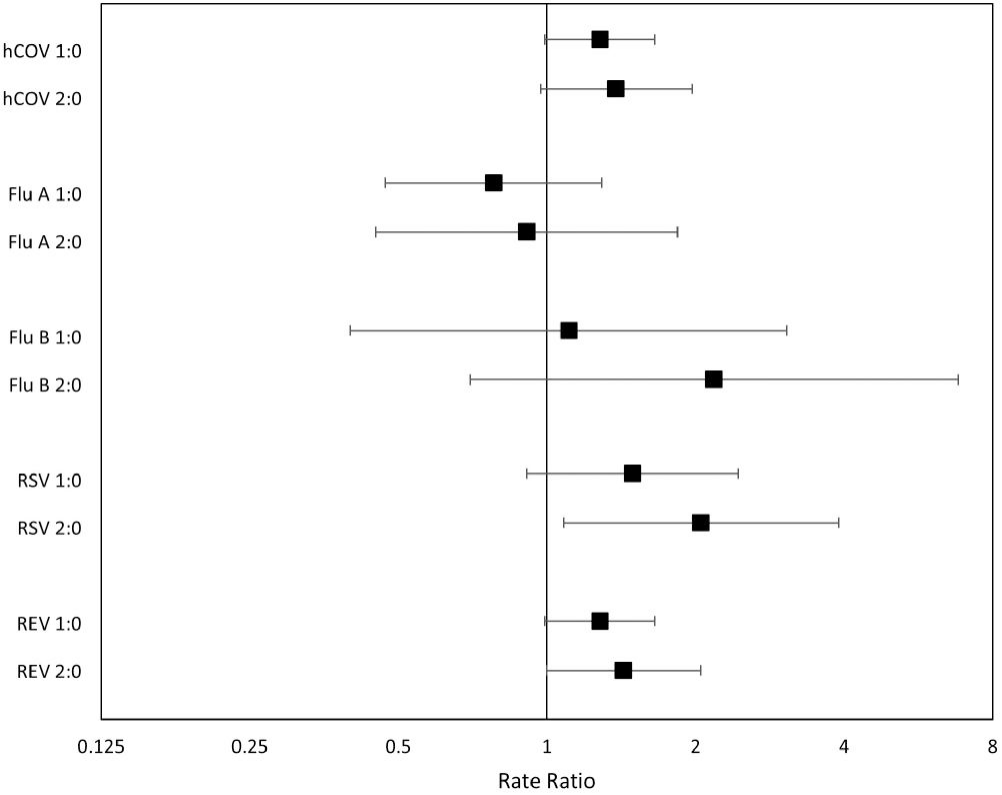
Adjusted incidence rate ratios (IRRs) for polymerase chain reaction (PCR)–detected viral respiratory infections with specific viruses. For each analysis, “1:0” compares 1 preschool-aged household contact (HHC) to zero, and “2:0” compares ≥2 preschool-aged HHCs to zero. IRRs were calculated using a mixed-effects Poisson regression model that accounted for clustering at the clinic level and was adjusted for age, sex, and occupational exposure risk. There were too few events for parainfluenza virus to run the model. Adenovirus and human metapneumovirus had very wide confidence intervals and are also excluded. Abbreviations: Flu A, influenza A; Flu B, influenza B; hCOV, endemic human coronaviruses; REV, human rhinovirus or enterovirus; RSV, respiratory syncytial virus.

The virus most associated with preschool-aged HHCs was RSV. The incidence rate of RSV PCR-VRIs for those with 1 and ≥2 preschool-aged HHCs was 49% greater (IRR, 1.49 [95% CI, .91–2.44]) and 105% greater (IRR, 2.05 [95% CI, 1.08–3.90]), respectively (*P* = .06 by LRT; Figure 3, Supplementary Table 4). Rhinovirus/enterovirus and endemic hCOVs were moderately associated with preschool-aged HHCs (*P* = .05 and *P* = .07, respectively, by LRT). Conversely, influenza A was not associated with having preschool-aged HHCs (IRR for 1 HHC, 0.78 [95% CI, .47–1.29]; IRR for ≥2 HHCs, 0.91 [95% CI, .45–1.84]; *P* = .61 by LRT). For the other respiratory viruses, there were too few events to make conclusions.

## DISCUSSION

In this cohort of HCPs working in outpatient settings, we found that exposure to preschool-aged children at home increased the risk of experiencing VRIs. The effect of preschool-aged HHCs was strongest for respiratory viruses that are more common in young children (RSV, rhinovirus/enterovirus, hCOVs [3, 24–26]), which has implications for mitigation strategies. Importantly, in this HCP population with high influenza vaccination rates, there was no additional risk for influenza infection associated with exposure to children in the home.

In this large cohort of HCPs, VRIs were identified using a structured symptom screening and testing process with a low rate of withdrawal. This systematic process enhanced the detection of VRIs in participants, for whom many infections are mild and do not require medical care. In addition, the use of a multiplex PCR assay provided for improved sensitivity to detect multiple respiratory viruses, which was important in a population that had high influenza vaccination coverage. Finally, the inclusion of HCPs with varied exposures and job functions, who served varied patient populations and practiced at geographically separated sites around the country over multiple years, improved the generalizability of these results.

The 4 sensitivity analyses were consistent, which supports the robustness of the findings. The first looked at the outcome of PCR-confirmed symptomatic illnesses. The second looked at the ITT cohort, which is less prone to selection bias. The third examined the subset of individuals who participated in >1 season and had their number of preschool-aged HHCs change at least once between seasons. Being compared to themselves between seasons, they may have had more similar measured and unmeasured characteristics, reducing the chances for unidentified confounders. The last analysis looked at the outcome of symptomatic illnesses, regardless of PCR testing.

These findings are concordant with a few other studies that link HHCs to VRIs in HCP. A prospective cohort of Canadian HCPs exposed to ill HHCs of any age were much more likely to develop VRIs (adjusted odds ratio, 7.0) [7], but the investigators did not assess whether there was a dose-dependent response by recording the number of HHCs as a risk factor. Studies have demonstrated that the risk of influenza infection in HCPs is 1.7- to 13.8-fold higher if living with children [15, 16]. Our study did not find that living with preschool-aged HHCs increased the risk for influenza, potentially explained by high influenza vaccination coverage among the participants, or because school-aged children pose a greater risk. A previous study using data from the ResPECT trial focusing only on endemic hCOV infections found that the number of preschool-aged household contacts, as a continuous variable, was associated with hCOV infection in univariable but not multivariable analysis [12]. The present study differs from this by categorizing together HCPs with ≥2 preschool-aged HHCs, but reached similar conclusions regarding the effect of preschool-aged HHCs on hCOV infection.

Compared to other studies, our study has several strengths. The primary outcome was a composite of infections with the most common respiratory viruses and did not focus exclusively on influenza. The outcome we used included asymptomatic infections, which may have otherwise been missed and may pose a risk for secondary transmission to others. However, some of the detections in asymptomatic individuals were likely from prolonged shedding following an acute infection. Additionally, rather than grouping all children together, we only looked at preschool-aged HHCs, which may pose the greatest risk for secondary viral transmission within households for certain noninfluenza viruses [17, 18]. Finally, by categorizing the number of preschool-aged HHCs into 3 groups, we were able to show a “dose-dependent effect” of preschool-aged HHCs, which strengthens the argument for causality.

The results of this study demonstrate that among HCPs, who generally are considered at elevated risk of occupational exposure to respiratory viruses, having young children at home is an independent risk factor for VRIs. This finding, while not surprising, implies that some VRIs among HCPs are acquired at nonoccupational settings. This may help explain why it can be challenging to assess the impact of workplace interventions in clinical studies of HCPs. More studies that measure all VRIs among HCPs, rather than isolating healthcare-acquired infections, will be needed to ultimately inform how to best prevent VRIs in HCPs, especially those with young children.

Furthermore, these findings have several implications for efforts to protect HCPs from VRIs and may be extrapolated to other adult populations. First, workplace interventions such as use of personal protective equipment are effective, but many VRIs in HCPs are not healthcare-acquired and are not preventable by workplace interventions. Further efforts to reduce VRIs in HCPs could target prevention of home exposures, which can be difficult to accomplish due to developmentally normal behaviors of infants and toddlers. In addition, there may be important public health implications for what mitigation strategies are implemented in childcare settings, such as restriction of sick individuals, improved hand hygiene, and mask wearing. Second, to reduce HCP presenteeism (working while infectious with a respiratory virus), not only should employees be encouraged to stay home while ill with symptoms of a respiratory illness, but nonpunitive sick leave policies or reassignment of roles should be considered.

This study has several limitations. The study population differs in characteristics from the general adult population, based on demographics and influenza vaccine coverage [27, 28]. Because ResPECT study participation within each clinic was optional, those who elected to not participate in the study may have had different characteristics than those who did, such as perceived low occupational risk of exposure. This may have led to an overestimate of the incidence rate of VRIs. Although only a small proportion of the participants were excluded due to missing data on HHCs, those who were excluded were different in several characteristics than those who were included, which may have slightly biased the results in either direction. The follow-up time for each HCP-season was determined using only the day of activation and the last day of follow-up, which assumes that all participants would have reported all symptomatic events over this time. Differential reporting of symptomatic events could bias the results of this study if such reporting were associated with number of HHCs and incidence of VRIs. The timing of the study in each year was specifically determined around influenza incidence and was not designed to capture epidemics of RSV and other respiratory viruses. This could have led to an underestimation of the rates of VRIs and an underestimation of the actual impact of preschool-aged HHCs. Because adults may be paucisymptomatic, missing VRIs in HCP-seasons with and without preschool-aged HHCs would bias the results toward the null. Although the original study was a randomized trial, the current study used groups that were nonrandomized with respect to preschool-aged HHCs and the effect estimates could be confounded. No strong confounders were identified in this study, but there may have been residual and unmeasured confounding such as childcare setting/environment, urban or rural residence, use of public transportation, and socioeconomic status. This study was not adequately powered to detect differential effects of exposures on different viruses.

We found that despite being exposed in the workplace, household exposures are likely a strong determinant of VRIs among HCPs. Still, there remain knowledge gaps on the specifics of respiratory virus transmission dynamics at home and in healthcare settings and on modifiable risk factors to prevent VRIs. The COVID-19 pandemic has taught us that the impact of VRIs in HCPs goes beyond the individuals infected. HCP VRIs and presenteeism or absenteeism due to VRIs are patient safety issues. Reducing VRIs in HCPs should remain a priority.

## Supplementary Material

ofad057_Supplementary_Data

## Supplementary Data

Supplementary materials are available at *Open Forum Infectious Diseases* online. Consisting of data provided by the authors to benefit the reader, the posted materials are not copyedited and are the sole responsibility of the authors, so questions or comments should be addressed to the corresponding author.

## Notes

***Prior publications.*** This is a secondary analysis of the Respiratory Protection Effectiveness Clinical Trial (ResPECT). There have been several publications to date on the primary results and secondary analyses of the ResPECT trial. Additionally, a thesis regarding these data was submitted in part for fulfillment of a Master of Science degree for Z. M. M. at the London School of Hygiene and Tropical Medicine, United Kingdom.

***Financial support.*** The ResPECT study was supported by the Centers for Disease Control and Prevention; the US Department of Veterans Affairs; and the Biomedical Advanced Research and Development Authority. C. W.-G. was supported by a Wellcome Intermediate Clinical Fellowship (201440/Z/16/Z).
