# Supplementary material for: Preschool-Aged Household Contacts as a Risk Factor for Viral Respiratory Infections in Healthcare Personnel
Source: Open Forum Infect Dis. 2023 Feb 8;10(2):ofad057. doi: 10.1093/ofid/ofad057 (PMC9942663; doi:10.1093/ofid/ofad057)
Supplement: ofad057_Supplementary_Data [file ofad057_supplementary_data.docx]

Supplemental Material

Contents

[Supplemental Methods 2](#_Toc125121274)

[Supplemental Results 4](#_Toc125121275)

[Supplemental Table 1 5](#_Toc125121276)

[Supplemental Table 2 6](#_Toc125121277)

[Supplemental Table 3 7](#_Toc125121278)

[Supplemental Table 4 8](#_Toc125121279)

# Supplemental Methods

A combined nylon tipped swab of the nares and pharynx was used for all specimen collections (FLOQSwabs UTM, Diagnostic Hybrids, Athens, OH). The respiratory specimens underwent automated extraction of nucleic acid (NorDiag Arrow, Roche, Indianapolis, IN) that was amplified via RT-PCR with a respiratory virus identification kit (PLEX-ID RVS 3.0, Abbott Molecular, Des Plaines, IL)

The ResPECT trial defined laboratory-detected respiratory infection as a secondary outcome of interest. This included any event with a positive RT-PCR test for any of the viruses studied (whether or not the participant was symptomatic) plus any season where an individual seroconverted their influenza hemagglutination inhibition antibody titer, timing the pre and post-season serum specimen collections to avoid the effects of pre-season vaccination (only when a PCR detected influenza A infection was not detected for that season). For the study presented in this article, an alternate but similar definition, polymerase chain rection detected viral respiratory infection (PCR-VRI) is used that ignores all influenza hemagglutination inhibition antibody titer results. This was done because the primary focus of this study was on all respiratory viruses, compared to the ResPECT trial where the primary focus was on influenza infection. Having the influenza inhibition antibody titers without any similar titers for the other viruses investigated would have biased the results more towards influenza. By ignoring these titers, all viruses in the study are handled the same way.

# Supplemental Results

In the entire ResPECT trial there were 57,414 weeks of follow-up over 5,180 participant-seasons with a total of 11,603 swabs for respiratory virus PCR testing collected. Of those, 3,468 (29.9%) were collected when the participant had symptoms of a respiratory infection and 8,135 (70.1%) were collected when the participant was asymptomatic. Not every symptomatic event qualified as an acute respiratory infection (ARI). There were a total 3,234 ARIs. Of those, 2,411 (74.5%) had a swab collected during the same calendar week as the ARI, while 1,057 (25.5%) did not have a swab collected during the same calendar week as the ARI.

| Supplemental Table 1. Comparison of participant-seasons with and without missing data on household contacts 0-5 years-old | | | | |
| --- | --- | --- | --- | --- |
| Exposure | Category | Not missing (N=4,476), no. (%) | Missing  (N=213), no. (%) | *P*^a^ |
| Sex | Female | 3,823 (85.4) | 180 (84.5) | 0.72 |
|  | Male | 653 (14.6) | 33 (15.5) |  |
| Age  (n=4,683) | 18-29 | 648 (14.5) | 27 (12.7) | <0.001 |
|  | 30-39 | 1,329 (29.7) | 46 (21.6) |  |
|  | 40-49 | 1,115 (24.9) | 41 (19.3) |  |
|  | 50-59 | 1,020 (22.8) | 72 (33.8) |  |
|  | 60+ | 358 (8.0) | 27 (12.7) |  |
| Race/Ethnicity | NH-White | 1,973 (44.1) | 53 (24.9) | <0.001 |
|  | NH-Black | 1,264 (28.2) | 55 (25.8) |  |
|  | Hispanic | 689 (15.4) | 53 (24.9) |  |
|  | Asian | 337 (7.5) | 36 (16.9) |  |
|  | Other | 213 (4.8) | 16 (7.5) |  |
| Smoker  (n=4,649) | Yes | 369 (8.3) | 19 (9.1) | 0.69 |
|  | No | 4,071 (91.7) | 190 (90.9) |  |
| Lung disease  (n=4,624) | Yes | 458 (10.3) | 19 (11.4) | 0.65 |
|  | No | 3,999 (89.7) | 148 (88.6) |  |
| Flu vaccine  (n=4,658) | Yes | 3,663 (82.3) | 138 (66.7) | <0.001 |
|  | No | 788 (17.7) | 69 (33.3) |  |
| Facial protection^b^ | MM | 2,355 (52.6) | 91 (42.7) | 0.005 |
|  | N95 | 2,121 (47.4) | 122 (57.3) |  |
| Patient-type | Pediatric | 1,011 (22.6) | 15 (7.0) | <0.001 |
|  | Adult | 2,428 (54.2) | 189 (88.7) |  |
|  | Both | 1,037 (23.3) | 9 (4.2) |  |
| Occupation risk^c^  (n=4,688) | High | 2,680 (59.9) | 135 (63.7) | 0.49 |
|  | Medium | 536 (12.0) | 21 (9.9) |  |
|  | Low | 1,260 (28.2) | 56 (26.4) |  |
| Year | 2011-12 | 531 (11.9) | 89 (41.8)^d^ | <0.001 |
|  | 2012-13 | 1,074 (24.0) |  |  |
|  | 2013-14 | 1,327 (30.0) | 52 (24.4) |  |
|  | 2014-15 | 1,544 (34.5) | 72 (33.8) |  |
| Site | Johns Hopkins Health System | 1,561 (34.9) | 0 | <0.001 |
|  | Denver Health Medical System | 929 (20.8) | 26 (12.2) |  |
|  | VA New York Harbor Healthcare System | 575 (12.9) | 184 (86.4) |  |
|  | The Michael E. DeBakey VA Medical Center | 257 (5.7) | 3 (1.4)^d^ |  |
|  | Washington DC VA Medical Center | 348 (7.8) |  |  |
|  | VA Eastern Colorado Healthcare System | 454 (10.1) |  |  |
|  | Children’s Hospital Colorado | 352 (7.9) |  |  |
| Those with missing data had a PCR-VRI rate of 1.3 per 100 participant-weeks. Those without missing data had a PCR-VRI rate of 2.0 per 100 participant-weeks  N is the number of healthcare personnel-seasons  a – calculated using chi-squared test  b – type of facial protective equipment assigned in the cluster randomized trial  c – occupational risk scale: high – direct patient contact with performance of high-risk procedures (intubation, airway suctioning, nebulizer treatments, nasopharyngeal aspiration), medium – direct patient contact without high-risk procedures, low – minimal patient contact  d – cells merged due to sparse values to protect participant identities  NH – non-Hispanic, MM – medical mask, N95 – N95 respirator | | | | |

| Supplemental Table 2. Multivariable adjustments of the association between preschool-aged household contacts and incidence of PCR-detected viral respiratory infections (N=4,470 HCP-seasons) | | | | |
| --- | --- | --- | --- | --- |
| Exposure | Category | Adjusted Rate ratio | *P*^a^ |  |
| No. HHC 0-5 years-old | 0 | 1.00 (ref) | --- |  |
|  | 1 | 1.22 (1.05-1.43) | 0.01 |  |
|  | 2+ | 1.35 (1.09-1.67) | 0.006 |  |
| Sex | Female | 1.00 (ref) | --- |  |
|  | Male | 0.89 (0.74-1.06) | 0.19 |  |
| Age (years) | 18 – 29 | 1.00 (ref) | --- |  |
|  | 30 – 39 | 0.84 (0.70-1.00) | 0.05 |  |
|  | 40 – 49 | 0.76 (0.63-0.91) | 0.004 |  |
|  | 50 – 59 | 0.69 (0.56-0.84) | <0.001 |  |
|  | 60+ | 0.71 (0.54-0.93) | 0.01 |  |
| Occupation risk | High | 1.15 (1.00-1.32) | 0.05 |  |
|  | Medium | 1.03 (0.84-1.27) | 0.77 |  |
|  | Low | 1.00 (ref) | --- |  |
| The adjusted model is a mixed-effects Poisson regression including all variables listed, accounting for clustering at the clinic level.  a *–* calculated by Wald test in mixed-effects model  HCP – healthcare personnel, HHC – household contacts, NH – non-Hispanic, MM – medical mask, N95 – N95 respirator | | | | |

| Supplemental Table 3. Sensitivity analyses assessing the association between preschool-aged household contacts and viral respiratory infections in healthcare personnel | | | | | | | | |  |
| --- | --- | --- | --- | --- | --- | --- | --- | --- | --- |
|  |  |  |  | Unadjusted | |  | Adjusted^a^ | | |
| Analysis | No. HHC 0-5 years | No. events | Follow-up time | Rate ratio  (95% CI) | *P*^b^ |  | Rate ratio (95% CI) | *P*^b^ | |
| Symptomatic PCR Confirmed  (N = 4,470) | 0 | 430 | 40,455 | 1.00 (ref) | 0.02 |  | 1.00 (ref) | 0.08 | |
|  | 1 | 127 | 9,407 | 1.27 (1.04-1.55) |  |  | 1.21 (0.99-1.49) |  | |
|  | 2+ | 54 | 3,743 | 1.35 (1.02-1.80) |  |  | 1.28 (0.96-1.72) |  | |
| ITT cohort  (N = 4,668) | 0 | 782 | 41,391 | 1.00 (ref) | <0.0001 |  | 1.00 (ref) | 0.003 | |
|  | 1 | 234 | 9,587 | 1.29 (1.12-1.50) |  |  | 1.22 (1.05-1.42) |  | |
|  | 2+ | 104 | 3,801 | 1.45 (1.18-1.78) |  |  | 1.36 (1.10-1.67) |  | |
| Changing number of HHC  (N = 644) | 0 | 52 | 3,080 | 1.00 (ref) | 0.15 |  | 1.00 (ref) | 0.16 | |
|  | 1 | 76 | 3,268 | 1.36 (0.95-1.94) |  |  | 1.36 (0.95-1.95) |  | |
|  | 2+ | 33 | 1,367 | 1.45 (0.93-2.26) |  |  | 1.42 (0.90-2.23) |  | |
| ARI | 0 | 2,182 | 40,455 | 1.00 (ref) | <0.0001 |  | 1.00 (ref) | 0.001 | |
| (N = 4,470) | 1 | 584 | 9,431 | 1.15 (1.04-1.26) |  |  | 1.11 (1.01-1.22) |  | |
|  | 2+ | 271 | 3,743 | 1.31 (1.15-1.49) |  |  | 1.26 (1.11-1.44) |  | |
| Symptomatic PCR Confirmed: symptomatic event along with detection of a respiratory virus by polymerase chain reaction  ITT cohort: all enrolled participants with at least one week of follow-up time  Changing number of HHC: who had their number of preschool-aged (0-5 years-old) HHC change at least once between seasons. There were 242 individuals in this group.  ARI – acute respiratory illness, regardless of the results of PCR testing  N is the number of HCP-seasons  Follow-up time is in weeks  All calculations account for clustering at the clinic level  a – adjusted for sex, age, and occupational risk using a mixed-effects Poisson regression model.  b – calculated by likelihood ratio test  HHC – household contacts, CI – confidence interval, ITT – intention to treat | | | | | | | | |  |

| Supplemental Table 4. Rate of virus-specific PCR-detected viral respiratory infections in healthcare personnel by number of preschool-aged household contacts (N = 4,470 HCP-seasons) | | | | | | | |
| --- | --- | --- | --- | --- | --- | --- | --- |
|  |  |  | Unadjusted | |  | Adjusted^a^ | |
| Virus | No. HHC 0-5 years | Incidence rate (95% CI)^b^ | Rate ratio (95% CI) | *P^c^* |  | Rate ratio (95% CI) | *P^c^* |
| *ADV* |  |  |  |  |  |  |  |
|  | 0 | 0.03 (0.02-0.06) | 1.00 (ref) | 0.31 |  | 1.00 (ref) | 0.25 |
|  | 1 | 0.01 (0.00-0.08) | 0.31 (0.04-2.34) |  |  | 0.25 (0.03-1.94) |  |
|  | 2+ | 0.05 (0.01-0.21) | 1.54 (0.35-6.79) |  |  | 1.12 (0.25-5.10) |  |
| *hCOV* |  |  |  |  |  |  |  |
|  | 0 | 0.63 (0.56-0.72) | 1.00 (ref) | 0.005 |  | 1.00 (ref) | 0.07 |
|  | 1 | 0.88 (0.71-1.10) | 1.39 (1.09-1.78) |  |  | 1.28 (0.99-1.65) |  |
|  | 2+ | 0.98 (0.71-1.35) | 1.54 (1.09-2.18) |  |  | 1.38 (0.97-1.97) |  |
| *hMPV* |  |  |  |  |  |  |  |
|  | 0 | 0.06 (0.04-0.09) | 1.00 (ref) | 0.008 |  | 1.00 (ref) | 0.01 |
|  | 1 | 0.16 (0.09-0.27) | 2.68 (1.40-5.11) |  |  | 2.51 (1.27-4.95) |  |
|  | 2+ | 0.03 (0.00-0.19) | 0.45 (0.06-3.36) |  |  | 0.42 (0.06-3.16) |  |
| *Flu A* |  |  |  |  |  |  |  |
|  | 0 | 0.26 (0.21-0.31) | 1.00 (ref) | 0.64 |  | 1.00 (ref)^d^ | 0.61 |
|  | 1 | 0.20 (0.13-0.32) | 0.79 (0.49-1.30) |  |  | 0.78 (0.47-1.29) |  |
|  | 2+ | 0.24 (0.13-0.46) | 0.94 (0.48-1.86) |  |  | 0.91 (0.45-1.84) |  |
| *Flu B* |  |  |  |  |  |  |  |
|  | 0 | 0.06 (0.04-0.10) | 1.00 (ref) | 0.54 |  | 1.00 (ref)^d^ | 0.46 |
|  | 1 | 0.06 (0.02-0.14) | 0.90 (0.34-2.38) |  |  | 1.11 (0.40-3.06) |  |
|  | 2+ | 0.11 (0.04-0.31) | 1.85 (0.63-5.38) |  |  | 2.18 (0.70-6.81) |  |
| *PIV* |  |  |  |  |  |  |  |
|  | 0 | 0.03 (0.02-0.06) | 1.00 (ref) | 0.03 |  | n/a | n/a |
|  | 1 | 0 (0-inf) | 0 (0-inf) |  |  |  |  |
|  | 2+ | 0 (0-inf) | 0 (0-inf) |  |  |  |  |
| *RSV* |  |  |  |  |  |  |  |
|  | 0 | 0.18 (0.14-0.22) | 1.00 (ref) | 0.11 |  | 1.00 (ref) | 0.06 |
|  | 1 | 0.24 (0.16-0.37) | 1.39 (0.87-2.23) |  |  | 1.49 (0.91-2.44) |  |
|  | 2+ | 0.32 (0.18-0.56) | 1.83 (0.99-3.37) |  |  | 2.05 (1.08-3.90) |  |
| *REV* |  |  |  |  |  |  |  |
|  | 0 | 0.63 (0.56-0.71) | 1.00 (ref) | 0.006 |  | 1.00 (ref) | 0.05 |
|  | 1 | 0.88 (0.71-1.09) | 1.40 (1.09-1.79) |  |  | 1.28 (0.99-1.65) |  |
|  | 2+ | 0.96 (0.69-1.33) | 1.53 (1.08-2.16) |  |  | 1.43 (1.00-2.05) |  |
| Accounts for clustering at the clinic level using a mixed-effects Poisson regression model  a – adjusted for sex, age, and occupational exposure risk  b – per 100 person-weeks  c – calculated by likelihood ratio test  d –adjusted model also includes influenza vaccination status  HCP – healthcare personnel, PCR – polymerase chain reaction, ADV – adenovirus, hCOV – endemic human coronaviruses, MPV – human metapneumovirus, Flu A – influenza A, Flu B – influenza B, PIV – parainfluenza virus, RSV – respiratory syncytial virus, REV – rhinovirus or enterovirus | | | | | | | |
